# Long non-coding RNAs and microRNAs as crucial regulators in cardio-oncology

**DOI:** 10.1186/s13578-022-00757-y

**Published:** 2022-03-04

**Authors:** Sarath Babu Nukala, Jordan Jousma, Yoonje Cho, Won Hee Lee, Sang-Ging Ong

**Affiliations:** 1 Department of Pharmacology & Regenerative Medicine, The University of Illinois College of Medicine, 909 S Wolcott Ave, COMRB 4100, Chicago, IL 60612 USA; 2 Department of Basic Medical Sciences, University of Arizona College of Medicine, ABC-1 Building, 425 North 5th Street, Phoenix, AZ 85004 USA; 3 Division of Cardiology, Department of Medicine, The University of Illinois College of Medicine, 909 S Wolcott Ave, COMRB 4100, Chicago, IL 60612 USA

**Keywords:** Non-coding RNAs, LncRNAs, miRNAs, Cardiotoxicity, Cardio-oncology

## Abstract

Cancer is one of the leading causes of morbidity and mortality worldwide. Significant improvements in the modern era of anticancer therapeutic strategies have increased the survival rate of cancer patients. Unfortunately, cancer survivors have an increased risk of cardiovascular diseases, which is believed to result from anticancer therapies. The emergence of cardiovascular diseases among cancer survivors has served as the basis for establishing a novel field termed cardio-oncology. Cardio-oncology primarily focuses on investigating the underlying molecular mechanisms by which anticancer treatments lead to cardiovascular dysfunction and the development of novel cardioprotective strategies to counteract cardiotoxic effects of cancer therapies. Advances in genome biology have revealed that most of the genome is transcribed into non-coding RNAs (ncRNAs), which are recognized as being instrumental in cancer, cardiovascular health, and disease. Emerging studies have demonstrated that alterations of these ncRNAs have pathophysiological roles in multiple diseases in humans. As it relates to cardio-oncology, though, there is limited knowledge of the role of ncRNAs. In the present review, we summarize the up-to-date knowledge regarding the roles of long non-coding RNAs (lncRNAs) and microRNAs (miRNAs) in cancer therapy-induced cardiotoxicities. Moreover, we also discuss prospective therapeutic strategies and the translational relevance of these ncRNAs.

## Introduction

**Fig. 1:**
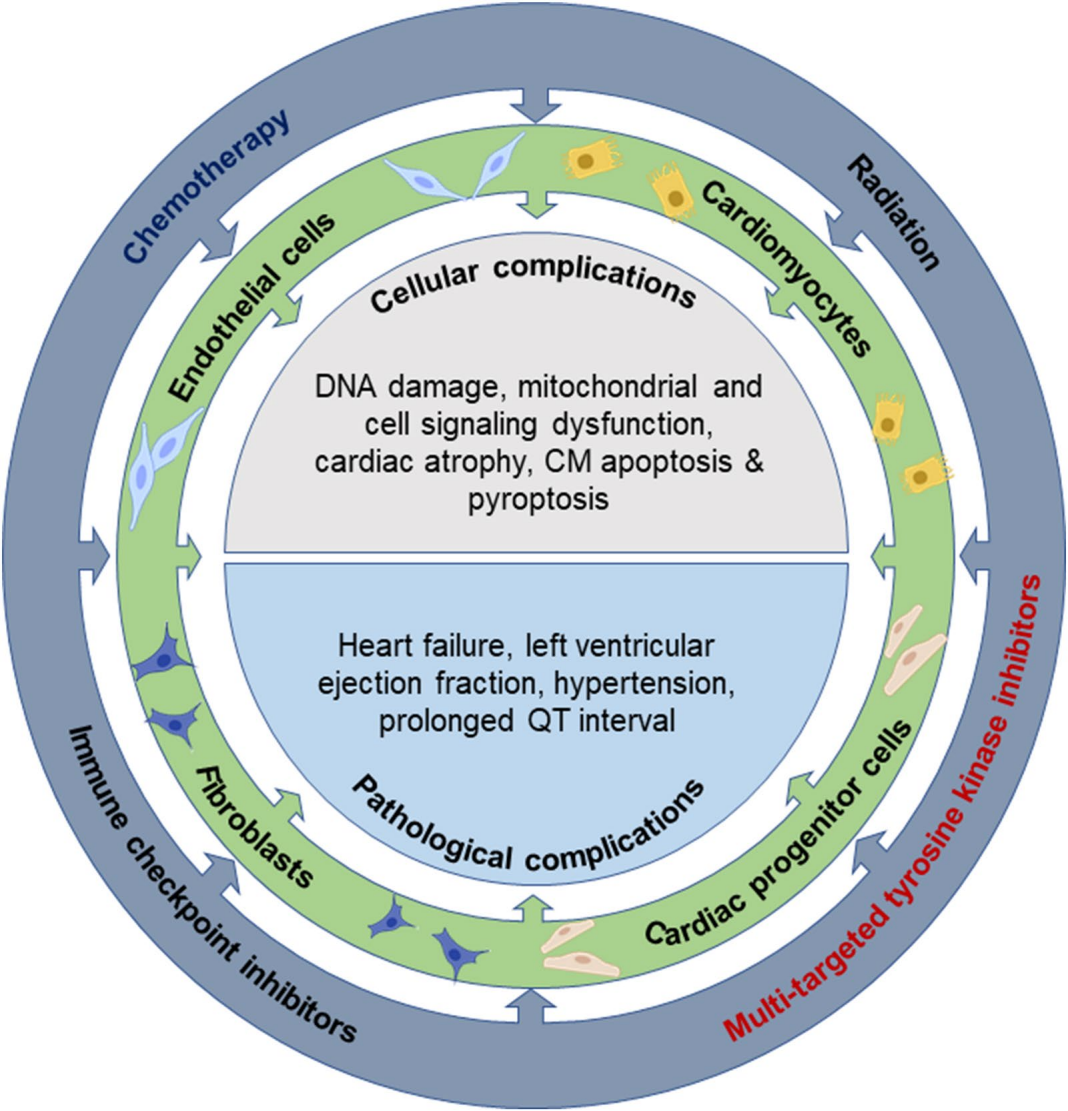
Cellular and pathological complications associated with cardiotoxicity induced by different cancer therapeutic strategies

Cancer is the second leading cause of death after cardiovascular disease (CVD), accounting for 0.6 million deaths in the United States alone in 2020 [1–3]. Despite having a seemingly different etiology, cancer and cardiac dysfunction appear intimately linked, especially when viewed in the context of cancer survivors. Cancer patients are known to frequently develop cardiovascular complications during and following treatment. The emergence of cardiovascular death as a leading cause of mortality among cancer survivors has given rise to the field termed ‘cardio-oncology.’ Cardio-oncology combines the efforts of both health care professionals and researchers with expertise in heart diseases and cancer. The overall aim of this inter-disciplinary collaboration is to address this emerging cardiovascular crisis observed among cancer survivors [4]. Nearly all cancer therapeutic strategies, including chemotherapies, immune checkpoint inhibitors (ICI), monoclonal antibody-mediated targeted therapies, and radiotherapies, are associated with mild to severe cardiovascular complications (Fig. 1) [5–10] The most frequently reported cancer therapy-induced cardiotoxicities are hypertension, thromboembolism, angiogenesis, QT interval prolongation, and heart failure [11–13]. The pathological drivers of cardiac dysfunction are related to the type of cancer, the anti-cancer therapy being used, and genetic determinants of disease susceptibility [14]. Cardio-oncology is in this sense at the forefront of an evolving field of medical sciences in that it recognizes an emergent health concern and is uniquely situated to implement principles related to personalized therapies.

Cardiotoxicity resulting from cancer therapies can be associated with defects at the molecular, structural, and functional levels of the heart. Anticancer agents induce cardiotoxic effects through multiple mechanisms, for instance, by interfering with DNA replication and repair mechanisms, increased production of reactive oxygen species (ROS), induction of non-specific immune responses, perturbations of electrophysiological signals, as well as mitochondrial dysfunction. These effects are present in multiple cell types within the cardiovascular system, including endothelial cells, ventricular and atrial cardiomyocytes (CMs), as well as fibroblasts [15–18]. While some of these effects are believed to be a direct result of the mechanism of action of the anti-cancer agent themselves, there are many instances whereby cardiac dysfunctions are driven by additional, unrecognized mechanisms. In these instances, there has been much interest in the functions of ncRNAs, which have been demonstrated to be responsible for cardiac dysfunction. Furthermore, experimental evidence has also shown that the targeting of these ncRNAs has the potential to attenuate the deleterious effects of various cancer agents. This has not only brought into focus the importance of ncRNAs in cardiovascular function but also demonstrated the therapeutic potential of ncRNAs in the context of cardio-oncology. Thus, there has been much interest in the field of cardio-oncology in the role of ncRNAs in mediating the effects of anticancer therapy-induced cardiotoxicity.

## Cardiotoxicity is associated with anticancer therapies

The advent of cancer therapies such as anthracyclines and tyrosine kinase inhibitors (TKIs) have heralded impressive improvements in long-term survivorship among cancer patients. Unfortunately, the cost of these benefits, in many cases, comes at the expense of the adverse effects upon the cardiovascular system. This has become increasingly apparent among cancer survivors [19, 20]. The anthracycline class of drugs is widely used to treat various types of cancers, including leukemias, lymphomas, breast, stomach, uterine, ovarian, bladder, and lung cancers. Within the class of anthracyclines is Doxorubicin (DOX), a broad-spectrum anti-tumor drug derived from *Streptomyces peucetius* var. *caesius*, which has a remarkable therapeutic effect on acute lymphoblastic leukemia, lung, and breast cancers [21]. Despite being one of the most extensively applied and studied chemotherapeutic agents, the clinical applications of DOX are hampered due to the severe cardiotoxic side effects [14, 22]. In a landmark study that exposed the cardiotoxic effects of DOX, it was recognized that among 2625 cancer patients undergoing treatment with anthracycline drugs, 157 patients developed heart failure. The resulting cardiac dysfunction was severe. Only 11% of the cohort of affected individuals would go on to benefit from existing heart failure therapies [23]. Studies such as this, as well as others have warranted the establishment of a new discipline of medicine known as cardio-oncology. The central focus of cardio-oncology is to improve patient outcomes among cancer survivors by addressing therapy-induced cardiac dysfunction. Within this focus is the need to investigate mechanisms by which these drugs exert harm to identify the novel druggable target.

Among the best-studied cancer drugs in this context is DOX. DOX-induced cardiotoxicity has been recognized as inducing a wide array of cardiovascular disorders. Among the most common is the development of cardiomyopathies, such as dilated cardiomyopathies and irreversible degenerative cardiomyopathies, which lead to heart failure [24, 25]. Additionally, DOX is also known to induce adverse electrophysiological remodeling leading to several different arrhythmias [26–28]. In the past two decades, researchers have extensively studied the clinical manifestations of DOX-induced cardiotoxicity. However, in-depth knowledge pertaining to DOX-induced cardiotoxicity has remained elusive. Literature has often suggested that underlying mechanisms related to DOX-induced cardiotoxicity are a result of on-target mechanisms of action of DOX, which involve acting as an intercalating agent, thus rendering CMs vulnerable to genotoxicity. More recently, though, it has been shown that these same intercalation mechanisms can also serve to displace histones, leading to a re-arrangement of the chromatin architecture, which may directly contribute to aberrant gene expression through epigenetic mechanisms [29]. Insights gained during the genomic revolutions have allowed for the mechanism by which a drug acts to be extended into uncharted territories of biological functions and reveals that the effects of pharmacological agents are often much more complex than previously believed. While some of the pathological consequences may be directly mediated through well-understood mechanisms of action, there are numerous other, poorly understood pathological consequences whose understanding might greatly benefit by exploring these areas. In the case of DOX, for instance, there have been numerous harmful effects which include accumulation of iron in mitochondria leading to ROS formation [30, 31], endoplasmic reticulum-mediated apoptosis [32, 33], lipid peroxidation [34], and dysregulation of intracellular calcium homeostasis [35, 36]. Other types of anthracycline drugs, including epirubicin [37, 38], daunorubicin [39], and idarubicin [40], are also known to cause cardiovascular complications. Improved understanding of the biological mode of actions of these anthracyclines will help to mitigate potential cardiovascular complications.

Small molecule TKIs are a newer class of targeted cancer drugs that inhibit or block the receptors of one or more tyrosine kinase enzymes. Multiple TKIs have been developed since the first Food and Drug Administration (FDA) approval for imatinib to treat chronic myeloid leukemia [41]. However, pre-clinical studies and post-marketing analysis demonstrated the association of severe cardiovascular complications with the use of TKIs [42]. Dasatinib, sorafenib, lapatinib, and sunitinib are among the most used TKIs. Dasatinib-induced cardiotoxicities include edema, fluid retention, pulmonary hypertension, and QT prolongation [43, 44]. The manifestations of sorafenib toxicity include hypertension, QT prolongation, and myocardial infarction [45–47]. Based on the existing literature, the possible underlying mechanisms for these side effects are related to the inhibition of various kinases, including B-RAF (serine/threonine-protein kinase B-Raf isoform 1), C-RAF (RAF proto-oncogene serine/threonine-protein kinase), C-KIT (KIT proto-oncogene, receptor tyrosine kinase), VEGFR (vascular endothelial growth factor receptor), PDGFRβ (platelet-derived growth factor receptor beta), and human ether-à-go-go-related gene (hERG) K+ channels [48, 49]. Clinical studies have also revealed the association of decreased left ventricular ejection fraction (LVEF) and QT prolongation as a side effect of lapatinib. Lapatinib which binds to the ErbB2 (Erb-B2 receptor tyrosine kinase), can result in activation of mitochondrial-induced apoptosis [46, 50]. Sunitinib-induced mitochondrial dysfunction leads to the release of cytochrome C and caspase-9, which initiate the mitochondrial apoptotic pathway [51]. Sunitinib, a multi-tyrosine kinase inhibitor, acts on VEGFR 1–3, c-KIT, PDGFR-α/β, RET (RET proto-oncogene), FLT3 (Fms related receptor tyrosine kinase 3), and CSF1R (colony-stimulating factor 1 receptor) [52]. Notable cardiotoxic effects associated with the use of sunitinib include decreased LVEF, chronic heart failure (CHF), and QT prolongation [51, 53].

Trastuzumab is a humanized monoclonal antibody targeted against HER2 (epidermal growth factor receptor 2), has been specifically recognized in the development of CHF and cardiac dysfunction [54, 55]. Trastuzumab alters the cell survival pathways of CMs by decreasing the expression of NRG-1 (neuregulin-1), resulting in the activation of FAK (focal adhesion kinases), PI3K/AKT (phosphoinositide 3-kinase/protein kinase B), and MAPK (mitogen-activated protein kinase) pathways [56]. Increased risk of cardiomyopathy is associated with trastuzumab when combined with anthracycline chemotherapy [57].

More recently, ICI and targeted immunotherapies have become increasingly popular as therapeutic options in the treatment of various cancers. This development has coincided with a steadily increasing prevalence of cardiovascular disease and death among cancer survivors. The mechanism of action of ICI involves the blockage of checkpoint proteins from binding with their corresponding partner proteins. This leads to the inhibition of an ‘off’ signal, resulting in the activation of T-cells to kill cancer cells. The list of ICIs also includes ipilimumab, which blocks the immune checkpoint molecule CTLA-4 (cytotoxic t-lymphocyte-associated protein 4); nivolumab and pembrolizumab, which target PD-1 (programmed cell death protein 1); atezolizumab, avelumab, durvalumab, and cemiplimab, which act against PD-L1 (programmed death-ligand 1). Although the cardiotoxicity associated with ICI therapy is believed to be lower than that which is observed with chemotherapy, it has only been recently introduced into the clinical setting, so the emergence of cardiovascular dysfunction among cancer survivors may continue to increase over time [58]. It is therefore important to the field of cardio-oncology to maintain surveillance over this population of patients being treated with ICI to assess for emerging cardiovascular disorders. Myocarditis, takotsubo syndrome, acute coronary syndrome, and peripheral diseases have recently been reported with increasing frequency among ICI patients [59, 60].

Chimeric antigen receptor (CAR) T-cell immunotherapy is an exciting breakthrough cancer treatment. The mechanism of action of this therapy involves the enhancement of T-cell function by adhering to a specificity defined with CARs [61–63]. Normal T-cells collected from cancer patients are infected with the modified virus, which can transfer cancer-targeted genetic information to the T-cell genome, which dictates the expression of newly synthesized CAR protein on the altered T-cell surface. Following ex vivo proliferation, these CAR T-cells are reinfused into cancer patients who have gone through cytotoxic lymphodepletion. The modified CAR T-cell implants and multiplies in the patient, resulting in targeted cancer cell apoptosis [64]. CD19-directed (axicabtagene, brexucabtagene, tisagenlecleucel) CAR T-cell therapy has been approved by the FDA as the first treatment for adults with advanced B-cell lymphoma and children with acute lymphocytic leukemia [65]. However, the side effect of CAR T-cell therapy is accompanied by hemodynamic instability and cytokine release syndrome (CRS), which is associated with adverse cardiac events, including cardiomyopathy, arrhythmias, and heart failure [66, 67]. Although the pathophysiology of CAR T-cell therapy-induced cardiovascular complications is not entirely understood yet, the potential underlying mechanism appears similar to that of cardiomyopathy associated with sepsis [68]. Another mechanism may be cross-reactivity with unrelated peptides expressed by normal tissue, leading to adverse cardiac events.

Arsenic trioxide (As_2_O_3_), a common component in traditional Chinese medicine, was initially regarded as a promising anticancer component used to treat acute promyelocytic leukemia, lung cancer, cervical cancer, and other malignant tumors [69–71]. Recent studies which recognize the cardiotoxic effects of As_2_O_3_ have halted its widespread use in clinical settings [72, 73]. Radiation therapy, one of the key strategies in treating several cancers, also carries a high risk of developing cardiovascular side effects, including myocardial fibrosis, pericarditis, congestive heart failure, acute coronary syndrome, myocarditis, and cardiomyopathy [74–77]. To date, there is no protective agent available for minimizing radiation therapy-associated cardiotoxicity except for reducing the radiation dose.

Proteasome inhibitors (PIs) are a new class of drugs that block the activity of proteasomes, developed for the treatment of multiple myeloma and mantle cell lymphoma [78]. Multiple PIs such as carfilzomib (Kyprolis) and ixazomib (Ninlaro) have been developed since the FDA approved Bortezomib (Velcade) in 2003 as the first-ever cancer treatment of this class of drugs [79]. Although the primary mechanism of action of PIs is associated with inhibition of proteasome activity, the precise downstream pathways that lead to the death of cancer cells remain unclear. Proteosome inhibition may prevent the degradation of p53 protein [80], allowing activation of programmed cell death in neoplastic cells. Proteosome inhibition in myeloma cells induces unfolded protein response (UPR) in endoplasmic reticulum leading to the activation of apoptotic events [81]. Similarly, inhibition of proteasomal-dependent protein turnover of sarcomeres in CMs leads to abnormal ubiquitinated protein accumulation, resulting in apoptosis and cell death [82]. Recent studies reported the association of congestive heart failure, hypertension, and arrhythmias as a result of PIs use [83]. Although there is evidence that all three FDA-approved PIs, bortezomib, carfilzomib, and ixazomib, are known to cause cardiovascular complications, the highest rate of cardiotoxicity allied with carfilzomib [83]. Heart failure [84], ischemic heart disease [85], complete heart block [86], and other complications have been reported with the use of bortezomib, which binds reversibly to β5 and β5i subunits of the immunoproteasome [87]. Arrhythmias, heart failure, ischemic heart disease, cardiomyopathy, pulmonary hypertension have been reported with the use of carfilzomib, which binds irreversibly to β5 and β5i subunits [88]. Like bortezomib, ixazomib also binds reversibly to β5 and β5i subunits along with β1 and β2 subunits [82, 87]. A study reported that ixazomib might induce cardiotoxicity in a similar way to other PIs suggesting a potential adverse class effect [89].

Traditional chemotherapeutic agents are cytotoxic, primarily affecting cell division by interfering with protein synthesis, DNA, RNA, or macromolecular synthesis [90]. Depending on the mechanism of action, they can be classified as alkylating agents, which produce unstable alkyl groups reacting with nucleophilic targets on proteins and nucleic acids. For instance, ifosfamide, busulfan, cisplatin, cyclophosphamide, chlormethine, carmustine, and mitomycin come under the class of alkylating agents which have been reported to induce cardiotoxicity [91]. Antimetabolites class of drugs affects multiple cellular pathways required for RNA and DNA synthesis. Mitotic inhibitors such as vinblastine or vincristine interfere with spindle assembly in mitosis. Antimicrotubule agents such as paclitaxel [92], etoposide [93], teniposide, vinca alkaloids have also been reported to induce cardiovascular complications [91].

ncRNAs play a vital role in maintaining several biological processes in both cancer and cardiovascular diseases [94–98]. Cancer therapeutic agents may induce abnormal changes in the expression of ncRNAs in cardiac and vascular cells resulting in the development of adverse effects in cardiovascular homeostasis. While the mechanisms of actions of these commercially available drugs are well understood as it relates to cancer, how these drugs contribute to the dysregulation of ncRNAs which might contribute to cardiac dysfunction, remains poorly understood. Thus, understanding the role of ncRNAs during cancer therapy-induced cardiotoxicity has become a focus among scientists in the field of cardio-oncology.

## ncRNAs

Recent studies have revealed that approximately 90% of the human genome is transcribed into ncRNAs [99, 100]. These ncRNAs include transfer RNAs (tRNAs), ribosomal RNAs (rRNAs), miRNAs, small interfering RNAs (siRNAs), piwi-interacting RNAs (piRNAs), small nucleolar RNAs (snoRNAs), small nuclear RNAs (snRNAs), extracellular (exRNAs), small conditional (scRNAs), lncRNAs, and circular RNAs (circRNAs). Previously, ncRNAs were considered to be non-functional byproducts of genetic information transfer from DNA to protein or transcriptional noise. However, many studies have shown that genetic knockout of specific ncRNA is lethal, which underscores the importance of these molecules in development and health [101]. While this suggests that the functional transcriptome is much larger than previously believed, it should be noted that most of the ncRNA lack conservation across mammalian species. Furthermore, while tens of thousands of ncRNA have been annotated, many of these transcripts appear to be very lowly expressed, sometimes only one copy per cell [102]. Further studies using single-cell RNA sequencing and primary cell types might better resolve these caveats which exist when evaluating bulk RNA sequencing data. Thousands of ncRNAs are identified in humans, mice (Fig. 3A), and other species. Due to the lack of evolutionary conservation, low expression levels of many of these annotations, and lack of data from primary cell lineages, the functional significance of ncRNAs has been disputed. To describe the level of conservation across mammalian species, we performed orthologous gene function analysis to check the percentages of matched annotations between different lineages using a reference list of human noncoding RNAs obtained from the HGNC (HUGO Gene Nomenclature Committee) data repository (Fig. 3B). Crucial roles for ncRNAs have been described during developmental as well as physiological and pathological states. ncRNA-mediated regulation has been observed in nearly all domains of biological function and is particularly well recognized in epigenetic, posttranscriptional, and translational aspects of gene regulation. Herein, we specifically address differentially regulated lncRNAs and miRNAs of interest to the field of cardio-oncology and provide an up-to-date review on their prospective and consequential influence in the field.

### lncRNAs

**Fig. 2:**
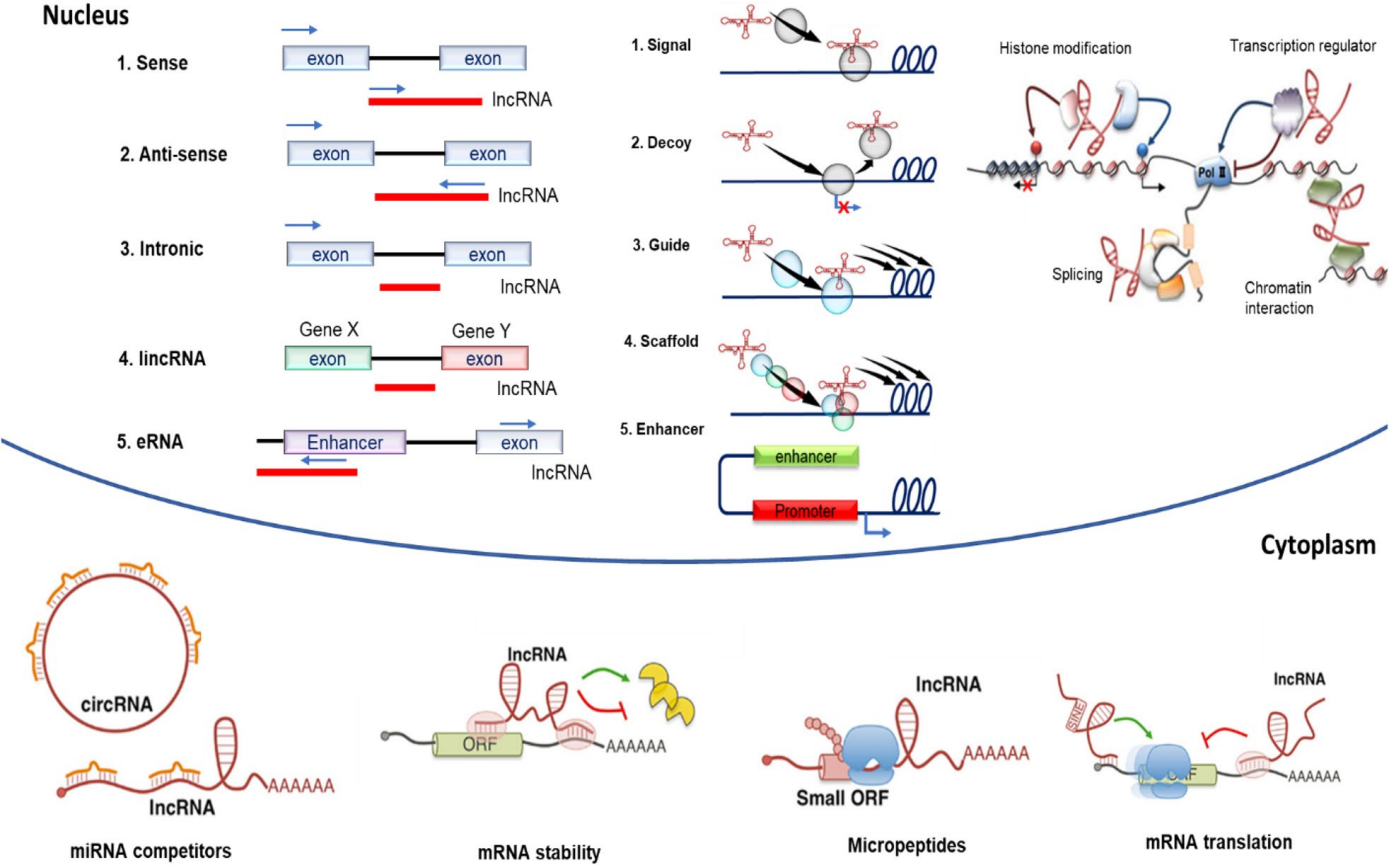
Classification, localization, and functions of lncRNAs

lncRNAs are a subset of ncRNAs, which contain over 200 nucleotides and resemble messenger RNA (mRNA) in the sense that they have both a 5′ cap and a 3′ polyadenylation and are capable of being spliced. While historically regarded as ‘junk DNA’ because they are not translated into protein products, new insights which recognize the impact of ncRNAs in regulating cardiovascular function have contributed to the evolution of biological understanding related to these molecules [103]. lncRNAs can be intronic, bidirectional, antisense, and sense-overlapping (Fig. 2) [104–107]. lncRNAs are expressed at low levels under physiological conditions since they are transcribed from promoters with low CpG dinucleotides. However, due to the modulation of chromatin states, lncRNAs expression can be aberrantly expressed. The expression of lncRNAs can be regulated in a tissue, cell-type, and disease-specific manner [108, 109]. In several pathological conditions, the presence of lncRNAs has been detected in body fluids, including blood, cerebrospinal fluid, and urine [110–114], suggesting potential utility as biomarkers with either diagnostic or prognostic value. Studies also reported that lncRNAs are encapsulated in exosomes and apoptotic bodies, usually bound to RNA-binding proteins [115, 116]. Moreover, selected lncRNAs are resistant to RNase degradation [117, 118].

The functions of lncRNAs associate with, and often depend on, subcellular localization (Fig. 2). lncRNAs regulate gene expression at the transcriptional level in the nucleus and the post-transcriptional level in the cytoplasm [119]. Nuclear lncRNAs interact with histone remodeling complexes to facilitate condensation or decondensation of the chromatin architecture. Additionally, they also interact with transcription factors to regulate gene expression by acting as a scaffold for proteins involved in transcription complexes [119]. For instance, lncRNAs MALAT 1 (metastasis-associated lung adenocarcinoma transcript 1), H19 (H19 imprinted maternally expressed transcript), and MEG 3 (maternally expressed 3) play a key role in cell cycle regulation through their interactions with p21 or P53 [120, 121]. However, lncRNAs promoter of PANDAR (promoter-CDKN1A antisense DNA damage activated RNA), lincRNA-p21 (long intergenic non-coding RNA-p21), RP11-467J12.4, and PINT (long intergenic non-protein coding RNA P53 induced transcript) are induced by p53 [121, 122]. Cytoplasmic lncRNAs can control mRNA stability, act as an assembly site for the RNP complex, or determine modifications of the cytoplasmic proteins [123]. Some cytoplasmic lncRNAs can also potentially translate into micropeptides [124]. These micropeptides have been reported to be involved in key biological mechanisms in various species [124–126], although this remains controversial and is an ongoing matter of investigation [124].

Various reports have described the regulatory interactions between lncRNAs and miRNAs [127–131]. This regulation can happen in two aspects. (1) Regulation of miRNAs by lncRNAs: lncRNAs can serve as precursors of miRNAs and, as such, are directly related to the function of miRNAs. lncRNAs are also important determinants of the functions of protein complexes that control gene expression. This is evident in complexes that regulate histone acetylation and transcription factor binding complexes. Finally, lncRNAs can act as a sponge of miRNAs, thereby inhibiting the degradation of mRNAs targeted by miRNAs. (2) Regulation of lncRNAs by miRNAs: miRNAs are known to regulate the expression of lncRNA genes through epigenetic mechanisms, involving DNA methylation and structural modifications to the chromatin [132]. For instance, miRNA-29 upregulates the expression of lncRNA MEG3 through the inhibition of DNA methyltransferase activity, resulting in a reduction of methylation of MEG3 promoter in hepatocellular cancer [121]. Additionally, miRNAs can degrade lncRNAs in an argonaute-dependent manner. miRNA binding to target lncRNAs within the 3′UTR are recognized by the RNA-induced silencing complex (RISC), which will lead to blockage of the ribosomal machinery or induce mRNA degradation, resulting in the silencing of gene expression [121].

Recent studies have demonstrated that lncRNAs have an essential role in the progression of cancer and cardiovascular disorders. Overall, the mechanistic basis involved in these events are diverse, involving alterations in the expression of genes and proteins involved in numerous signaling pathways and affecting multiple cell types of the cardiovascular system. In many instances, though, the involvement of mitochondrial dysfunction has been reported. In CMs, mitochondrial dysfunction, mainly increased production of ROS, has been reported to impair cellular processes such as cell proliferation, migration invasion, cell cycle, apoptosis and is even believed to contribute to drug resistance [103, 109, 133, 134]. The dysregulation of lncRNAs can lead to the progression of several cardiac diseases, including cardiomyopathies, congenital heart disease, cardiac hypertrophy, heart failure, coronary artery disease, and myocardial reperfusion injury [135–138]. lncRNAs are found ubiquitously within the pathology of cancer and cardiovascular disease, yet relatively little is known about them. Thus, this area of biology is amenable for the development of novel therapeutic strategies. In the emerging field of cardio-oncology, the roles of lncRNAs in cancer therapy-induced cardiotoxicity are increasingly being noted (Table 1).

**Table 1:** lncRNAs involved in cancer therapy-induced cardiotoxicity

lncRNA	Orthologs	Type	Drug	Expression	Targets	Cell type	Biological effect	Ref.
CMDL-1	*Rattus norvegicus*	Unknown	DOX	Down	Drp-1	CMs	Mitochondrial fission and apoptosis	[139]
SOX2-OT	*Homo sapiens* *Mus musculus*	Overlapping	DOX	Up	miR-942-5p	CMs	apoptosis	[231]
HOXB-AS3	*Homo sapiens*	Antisense	DOX	Up	miRNA-875-3p	CMs	Protects CMs	[350]
NEAT1	*Homo sapiens Mus musculus*	Intergenic	As_2_O_3_	Up	miR-124/NF-κB	CMs	Protects CMs	[149]
MALAT1/NEAT2	*Homo sapiens Mus musculus*	Intergenic	DOX	Up	miR-92a-3p/ATG4a miR-144–39	CMs	Mitochondrial metabolism & autophagy	[201, 205]
lincRNA-p21	*Homo sapiens Mus musculus*	Intergenic	DOX	Up	Wnt/β-catenin	CMs	silencing lincRNA-p21 effectively protects against DOX cardiotoxicity by regulating the Wnt/β-catenin signaling pathway and decreasing oxidant stress	[351]
NEAT1	*Homo sapiens Mus musculus*	Intergenic	DOX	Up	let-7f-2-3p	CMs	Attenuated cardiotoxicity via XPO1-mediated HAX-1 nuclear export	[151]
FOXC2-AS1	*Homo sapiens*	Antisense	DOX	Up	WISP1	CMs	Promoted DOX resistance and reduces the DOX-induced CM injury	[352]
PVT1	*Homo sapiens Mus musculus*	Intergenic	DOX	Down	miR-187-3p	CMs	Decreased the apoptosis of CMs	[353]
NEAT1	*Homo sapiens Mus musculus*	Intergenic	DOX	Up	miR-221-3p	CM and exosome^MIF^	Exosomal LncRNA–NEAT1 derived from MIF-treated mesenchymal stem cells protected	[153]
KCNQ1OT1	*Homo sapiens Mus musculus*	Antisense	As_2_O_3_	Down	Kcnq1	In vivo: mouse In vitro: CM	QT interval prolongation	[354]
SNHG1	*Homo sapiens*	Intergenic	DOX	Overexpression	miR-195/Bcl-2	CMs	Protected the CMs from DOX toxicity	[355]
LINC00339	*Homo sapiens*	Intergenic	DOX	Knockdown	miR-484	CMs	Improved cells proliferation activity and reduced CM apoptosis through miR-484 axis	[356]
CHRF	*Mus musculus*	Intronic	DOX	Knockdown	TGF-β1	CMs	Improved DOX-induced heart failure by regulating TGF-β1	[246]
TINCR	*Homo sapiens Mus musculus* *Rattus norvegicus*	Intergenic	DOX	Knockdown	NLRP3, IGF2BP1	CMs and heart tissues	Reversed the DOX-induced pyroptosis both in vitro and in vivo	[267]

#### Protective lncRNAs in cancer therapy-induced cardiotoxicity

##### CMDL-1 (cardiomyocyte mitochondrial dynamic-related lncRNA)

A recent study demonstrated that lncRNA CMDL-1 was significantly downregulated in DOX-treated CMs of rats [139]. Overexpressing of CMDL-1 attenuated DOX-induced mitochondrial fission and apoptosis in CMs by enhancing phosphorylation of dynamin-related protein 1 (Drp 1) at the S637 residue and inhibiting the Drp1 translocation to mitochondria. The full length of CMDL-1 is not highly conserved among species. However, this study provides evidence for the role of lncRNAs in posttranslational mechanisms [139]. In our knowledge, this is the first-ever study demonstrating the involvement of CMDL-1 in cancer therapy-induced cardiotoxicity, and therefore, more experiments need to be performed to characterize the lncRNA CMDL-1 and its therapeutic potential in reducing cardiotoxicity.

##### NEAT1 (nuclear enriched abundant transcript 1/nuclear paraspeckle assembly transcript 1)

The lncRNA NEAT1 has been observed to have increased expression levels in multiple cancers, including colorectal, lung, esophageal, and liver cancers, and has also been recognized as a regulator of cardiovascular function. In DOX-resistant human gastric cancer cells, knockdown of NEAT1 promotes apoptosis of DOX-resistant cells [140]. As it relates to cardiovascular function, NEAT1 has been reported to be involved in mediating cardiac cell damage and has been observed to be dysregulated in patients suffering from myocardial infarction [141–147]. Interestingly, NEAT1 has also been shown to have a protective role against hypoxia/reoxygenation-induced CM injury through the regulation of miRNA-520a [148]. The functions of NEAT1 are primarily a consequence of its scaffolding capabilities, which allow for it to interact with chromatin regulators and transcription complexes, as well as the underlying sequence of NEAT1, which provide it with the ability to act as a decoy for various miRNAs.

Approaches which have been able to sustain the expression of NEAT1 after exposure to the anti-cancer drug have been successful in attenuating some adverse cardiovascular effects. For instance, overexpression of NEAT1 rescued the inhibitory effect of As_2_O_3_ on the proliferation of H9C2 cells [149]. Additionally, NEAT1 has been reported to normalize the expression of inflammatory genes upregulated by As_2_O_3_ in CMs, including IL-1β (interleukin-1β), IL-6 (interleukin 6), and TNF-α (tumor necrosis factor α). When exposed to As_2_O_3_ or hypoxic stress, H9C2 cells upregulate the expression of miR-124 [150]. NEAT1 is known to act as a decoy to miR-124, leading to its downregulation. Experimentally this mechanism has been shown to confer cardioprotection. When overexpressed in CMs, NEAT1 has been shown to reduce the expression of inflammatory markers resulting from As_2_O_3_ treatment by quenching miR-124 and subsequent downstream NFκB mediated events [149].

NEAT1 has also been recognized as mediating cardioprotection against DOX-induced cardiotoxicity by sponging the miRNA known as let-7f-2-3p [151]. Treatment of H9C2 CMs with DOX leads to reduced expression of NEAT1 and subsequent increases in let-7f-2-3p expression. The resulting increased expression of let 7f-2-3p allows this miRNA to negatively regulate the expression of XPO1 (exportin-1). When XPO1 expression is reduced nuclear export, functions are impaired. This results in the nuclear accumulation of HAX-1, an important regulator of numerous myocardial enzymes. This leads to impaired calcium handling and increased apoptosis. Overexpression of XPO1 can reverse these effects by restoring the nuclear export of HAX-1. Similarly, overexpression of NEAT1 diminished the DOX-induced increase in let-7f-2-3p expression leading to reduced cardiotoxicity [151]. The inhibition of let-7f-2-3p has also been shown to improve DOX-induced heart injury without affecting the antitumor efficacy in vivo. In endothelial cells, overexpression of XPO1 has also been reported to protect against angiotensin II-induced injury [152]. In response to DOX-induced cardiovascular injury, the NEAT1/let-7f-2-3p/XPO signaling axis, therefore, represents a valuable pathway to investigate in the context of cardio-oncology, which might offer therapeutic insights. Additional mechanisms of cardio-protection against DOX-induced toxicity involve NEAT1 inhibition of miR-221-3p and the activation of SIRT2 (sirtuin 2) in a cardioprotective context [153].

The lncRNAs present in the exosomes regulate the expression of genes in host cells via cell-to-cell interactions [154]. It has been reported that lncRNA NEAT1 was highly expressed in exosomes derived from mesenchymal stem cells (MSCs) treated with macrophage migration inhibitory factor (MIF). The functions of NEAT1 in this context serve an anti-apoptotic role via competitive endogenous RNA (ceRNA) activity towards miR-142-3p. The NEAT1/miR-142-3p axis mediated the effect of exosomes isolated from MIF-pretreated MSCs (exosome^MIF^) in protecting CMs from apoptosis. Likewise, the protection of CMs in vitro by exosome^MIF^ was eliminated by knockdown of NEAT1 expression in MSCs or by miR-142-3p overexpression in CMs, indicating the important role of NEAT1 in cardiovascular protection [155]. Furthermore, Zhuang et al*.* demonstrated that the exosomes delivering NEAT1 have a therapeutic effect in vivo against DOX-induced cardiotoxicity and apoptosis [151].

Several studies have reported that transcription factors such as heat shock transcription factor 1 (HSF1) [156], hypoxia-inducible factor (HIF)-2 [157, 158], RUNX1 [159], and signal transducer and activator of transcription 3 (STAT3)/nuclear factor kappa-light-chain-enhancer of activated B cells (NF-κB) [160] bind to the promoter of NEAT1 and induce its expression. On the other hand, the downregulation of NEAT1 was regulated by breast cancer type 1 susceptibility protein (BRCA1) [161] and DNA methylation [162]. Studies have shown that HSF1 stimulates transcription of protein-coding genes specifically through binding with heat shock elements (HSEs) and repeated inverts of *n*GAA*n*, where “*n*” is any nucleotide, in the upstream regulatory regions of its target genes [163]. Similarly, HSF1 binds within three HSE regions located in the promoter region of lncRNA NEAT1 that is highly conserved among vertebrates [156]. Acetylation and deacetylation were reported to be involved in HIF2A mediated protein-coding gene expression [164]. In the case of lncRNA NEAT1, the binding of HIF2A was observed upstream of the promoter, suggesting a direct transcriptional control [157]. Runt-related transcription factor 1 (RUNX) regulates protein-coding genes through binding to Core Binding Factor Beta (CBF-β) to the Runt Homology Domain (RHD), nuclear matrix-targeting signal (NTMS), a conserved c-terminal domain, and VWRPY motif [165]. RUNX1 was reported to regulate the lncRNA NEAT1 by binding to the promoter region, indicating a direct transcriptional activity [159]. Studies reported that BRCA1 enhances transcription by recruiting the transcriptional machinery to targeted protein-coding genes. The role of BRCA1 in regulating gene transcription depends on its c-terminal portion, interacting with RNA polymerase II holoenzyme, and modulating the function of transcriptional activators [166]. Moreover, DNA methylation was observed to be an important determinant of NEAT1 expression [162]. DNA methylation regulates target gene expression by recruiting protein responsible for gene repression or preventing the binding of transcription factors to DNA [167].

##### KCNQ1OT1 (potassium voltage-gated channel subfamily Q member 1 overlapping transcript1)

KCNQ1OT1 is a highly conserved lncRNA, with nearly 80% of its genomic sequence identity being identical between humans and mice, which is particularly high for a non-coding gene [168]. KCNQ1OT1 belongs to a class of lncRNAs known as overlapping transcripts, which are most often involved in regulating their adjacent protein-coding gene. In the case of KCNQ1OT1, this corresponds to the well-studied potassium channel known as KCNQ1, which is known to be involved in the regulation of cardiovascular electrophysiological functions. The dysregulation of KCNQ1OT1 has been recognized in the pathology of electrophysiological disorders, including long QT syndrome, various arrhythmias, and the development of atherosclerotic plaques [169–172]. KCNQ1OT1 is frequently recognized as being overexpressed in patients suffering from different cancers, including breast, bladder, and tongue [169–172]. Additionally, anti-cancer treatments such as As_2_O_3_ can result in decreased expression of KCNQ1OT1 in cardiac tissue [170]. Mice treated with As_2_O_3_ have been recognized as having significant reductions in the expression of KCNQ1OT, which occurred in conjunction with the downregulation of the corresponding sense gene, describing a *cis*-regulatory mechanism of this lncRNA contributing to cardiac dysfunction. Experimentally, siRNA-mediated knockdown of KCNQ1OT1 has been shown to lead to an increased action potential duration (APD) in vitro, while similar approaches used in vivo resulted in Long QT Syndrome (LQTS) [170]. The downregulation of KCNQ1OT results in increased expression of miR-192-5p, which can increase cellular injury and apoptosis in H9C2 cells, an effect that can be reversed when KCNQ1OT is overexpressed.

β-Catenin was found to be regulating the transcription of lncRNA KCNQ1OT1 by direct binding to the proximal region of the imprinting control region within the KCNQ1OT1 promoter [173]. However, in the protein-coding genes, β-catenin was reported to bind with T-cell factor/lymphoid enhancer factor (TCF-LEF) transcription factor and induce target gene transcription, including CYCLIN D1, cMYC, PDK, MCT-1, AXIN2, and fibronectin [174]. A study reported that ubiquitously expressed mammalian transcription factor yin yang 1 (YY1) [175] was positively regulating the transcription of lncRNA KCNQ1OT1 by direct binding to the promoter. YY1 controls both transcription activation and repression in a contextualized manner [176].

##### MHRT (myosin heavy chain associated RNA transcripts)

The lncRNA MHRT originates from an intergenic region of the MYH7 (myosin heavy chain 7) gene loci. MHRT is recognized as being restricted to the nuclear fraction of CMs, where it regulates nucleosome remodeling by acting as a decoy to BRG1, a chromatin repressor complex. The resulting chromatin remodeling mediated by MHRT is linked to the dysregulation of genes involved in hypertrophic remodeling. Diminished expression levels of MHRT are frequently observed in cardiomyopathy. Thus, it is believed that maintaining the expression of MHRT might have a protective role against cancer therapy-induced hypertrophic remodeling. In DOX-treated hearts where cardiomyopathies develop, MHRT expression is observed to be downregulated [177, 178]. When expression of MHRT is maintained throughout DOX-treatment, pathological remodeling becomes attenuated. Obestatin, a peptide hormone protein encoded in the ghrelin gene, can lead to an upregulation of MHRT, which can confer cardioprotection against DOX-induced cardiomyopathy [177].

Mechanistically, MHRT mediated cardioprotection is believed to be mediated largely through epigenetics. In one instance, MHRT mediated upregulation of NRF2 (nuclear factor erythroid 2-related factor 2) gene, and protein expression has been observed to occur through acetylation of H3 [177]. The upregulation of NRF2 has previously been reported to have protective effects against the development of heart failure and adverse cardiac remodeling [179]. MHRT has also been reported to regulate HDAC5, which leads to altered levels of acetylation at the myocardin promoter [180]. MHRT expression has been shown to be negatively correlated with the expression of myocardin, a regulator of muscle growth. Overexpression of MHRT is associated with reduced acetylation at the promoter of myocardin in CMs, whereas knockdown of MHRT resulted in increased levels of acetylation at the myocardin promoter, effects which are proposed to result from interaction with HDAC5 [180]. Interestingly, myocardin has been shown to increase transcriptional activity of MHRT by forming a positive feedback loop via binding with the CarG box of MHRT promoter [180]. The myocardin family members of coactivators have been shown to activate genes responsible for cell migration, proliferation, and myogenesis through interacting with serum response factors (SRF) [181]. Association of myocardin with the histone acetyltransferase p300 increases the expression of SRF target genes, whereas its interaction with class II histone deacetylases represses the expression of SRF target genes [181, 182].

MHRT could serve as a potential therapeutic target to reduce cardiovascular diseases as well as cardiotoxicity induced by cancer therapeutic agents. However, transgenic mice models need to be generated to perform additional experiments to confirm that overexpression of MHRT might serve as a potential therapeutic target.

##### FOXC2-AS1 (forkhead box protein C2-Antisense RNA 1)

The lncRNA FOXC2-AS1 has been reported to promote DOX resistance in osteosarcoma in vivo and in vitro [183]. FOXC2-AS1 was found to be downregulated in heart tissues of mice in DOX-induced cardiotoxicity. Overexpression studies revealed that FOXC2-AS1 reduced CMs injury by increasing the expression of WISP1 (wnt1-inducible signaling pathway protein-1) to promote or sustain the activation of various cell survival pathways [184, 185]. However, these studies lack mechanistic insights regarding any possible intermediates that might involve the FOXC2-AS1 and WISP1-mediated cardioprotection against DOX-induced cardiotoxicity. Moreover, additional in vivo experiments are required to confirm the therapeutic potential of this lncRNA.

##### SNHG1 (small nucleolar RNA host gene 1)

The lncRNA SNHG1, located in human chromosome 11, is known to be differentially expressed in multiple types of cancers and CVDs [98, 186–191]. Zhang et al*.* reported that overexpression of SNHG1 mitigates the toxicity of oxidative stress in human CMs [187]. Recently, it has been shown that DOX downregulates the expression of SNHG1 in AC16 cells, leading to the increased expression of pro-apoptotic proteins BAX (BCL2 associated X, apoptosis regulator) and cleaved caspase-3 while decreasing the expression of anti-apoptotic protein BCL-2 (B-cell CLL/lymphoma 2). When overexpressed, though, SNGH1 was shown to reverse these effects, mainly by increasing cell viability, restoring the BCL2/BAX ratio, and decreasing the cleavage of caspase 3.

One of the mechanisms of action of SNHG1 involves acting as a ceRNA for miR-195. When the expression of miR-195 is increased, there is a reduction in the expression of BCL-2; likewise, when the miR-195 expression is reduced, there is a corresponding increase in the expression of BCL-2 [192]. SNHG1 has also been reported to reinforce anti-tumor properties of baicalein in the cervical cancer cell, affecting cell viability, migration, apoptosis, and tumor growth by regulating miR-3127-5p [193]. These studies on the cardioprotective property of SNHG1 and its role in cancer are based on in vitro data. Further in vivo experiments are needed to confirm the therapeutic potential of this lncRNA.

Specificity protein 1 (Sp1) [194], E2F Transcription Factor 1 (E2F1) [195], MYCN proto-oncogene, BHLH transcription factor (MYCN) [196] have shown to be positively regulated the expression of lncRNA SNHG1 through binding with upstream promoter region. The transcription factor Sp1 typically activates the transcription of cellular genes that has GC boxes in their promoter region. Moreover, the regulation of transcriptional activity of Sp1 has shown to be associated with post-translational modifications including phosphorylation, glycosylation, and acetylation [197]. The oncogenic transcription factor MYCN is known to act as an activator or repressor through heterodimerizing with Max to bind specific E-box DNA motifs (CANNTC), or recruiting corepressors, respectively [198]. Methyltransferase 3, n6-adenosine-methyltransferase complex catalytic subunit (METTL3)-mediated m6A modification has shown to be promoting the upregulation of SNHG1 by improving the stability of its RNA transcripts [199].

##### MALAT1 or NEAT2 (noncoding nuclear-enriched abundant transcript 2)

lncRNAs have emerged as a therapeutic target in reducing DOX-induced cardiac senescence [200]. The expression of MALAT1/NEAT2 was upregulated in DOX-treated CMs and in CMs treated with exosome^Hypoxia^ [201]. Silencing of MALAT1 in MSCs before treatment with hypoxia abolished the protective effect of exosome^Hypoxia^ in CMs, indicating that exosomes derived from MSCs^Hypoxia^ exerted a therapeutic effect partially mediated through direct transfer of MALAT1. miR-92a-3p has a binding site in the sequence of MALAT1. DOX-treated CMs showed increased levels of miR-92a-3p. However, the expression level of miR-92a-3p in DOX-treated CMs is substantially reduced when exosome^Hypoxia^ were added, while MALAT1 was highly expressed, indicating that MALAT1 directly inhibits mi-92a-3p against DOX-induced senescence. ATG4a was identified as a downstream target of miR-92a-3p. Thus, the exosomal transfer of lncRNA-MALAT1/miR-92a-3p and activation of ATG4a represents a pathway by which protection against DOX-induced cellular senescence can be achieved.

DOX-treatment or MALAT1 knockdown/miR-92a-3p overexpression mediated silencing of ATG4a resulting in increased the expression of FABP 3 and 4 (fatty acid–binding proteins 3 and 4) and MTFP1 (mitochondrial fission process 1) while decreasing the expression of Cox4i2 (cytochrome C oxidase subunit 4I2), HSPA1A (heat shock protein family A member 1A) and ATP1B2 (ATPase Na^+^/K^+^ transporting subunit beta 2) in CMs [201]. Previous studies also reported the effect of miR-92a-3p in impairing the metabolism of CM and autophagy by targeting ATG4a [202]. Overexpression of ATG4a benefits the heart during the ischemia/reperfusion process [203].

The lncRNA-MALAT1 has been linked to several kinds of human tumors and recognized as a prognostic biomarker for lung cancer metastasis. LncRNA-MALAT1 promotes CM apoptosis following myocardial infarction by targeting miR-144-39 [204, 205]. Reduced levels of MALAT1 have also been shown to augment atherosclerotic lesion formation in mice [206, 207]. In regard to potential therapeutic roles, MALAT1 has been recognized as helping address cardiac damage, by regulating hypoxia-inducible factors [208, 209]. Genetic variation (rs619586AG/GG genotype) of MALAT1 is associated with reduced risk for coronary atherosclerotic disease [210]. All these observations suggest that targeting MALAT1 might be a promising therapeutic strategy in reducing the cardiovascular toxic effects of anticancer drugs.

The transcription factors such as SP1 [211], SP3 [212], and c-MYC l [213] have been reported to positively correlate with MALAT1 through binding with promoter. In protein-coding genes, the binding of SP1 to DNA elements is mediated through the zinc finger domain which can recruit basal transcription machinery, while other domains facilitate interactions with chromatin remodeling complex to promote transcription [213]. HIF1/2A are also known to activate the expression of MALAT1 under hypoxic conditions [214, 215] by binding the hypoxia response element (HRE). β-Catenin and TCF/LEF have also been identified as downstream regulators protocadherin-10 (PCDH10)-Wnt signaling which regulates MALAT1 expression [216]. NRF1 and MALAT1 participate in a positive regulatory loop whereby MALAT1 mediates epigenetic silencing of kelch like ECH associated protein 1 (KEAP1), a negative regulator of NRF1, which stabilizes the expression of NRF1 and allows for increased NRF1 binding within the promoter of MALAT and subsequent increases in MALAT1 expression [217, 218]. Yes-associated protein 1 (YAP1) also positively regulates of MALAT1 in a β-catenin dependent fashion [219]. The histone remodeling complex, JMJD1A has also been recognized as integrating various upstream inputs, such as hypoxia [220] and cancer [221] which contribute to the regulation of MALAT1 expression. Negative regulation of MALAT1 is mediated through P53 which binds to the MALAT1 promoter, preventing Pol II binding [222]. Similarly, SRY-Box transcription factor 17 (SOX17) suppresses MALAT1 expression by binding to the promoter [223].

Other protective lncRNAs in reducing cardiac injury: The lncRNA UCA1 (urothelial cancer associated 1) decreased hypoxia and glucose deprivation-induced H9C2 injury in CMs by downregulating the expression of miR-122 [224]. Similarly, lncRNA TUG1 (taurine up-regulated 1) protected CMs from ischemia–reperfusion injury by downregulating HMGB1 (high mobility group box 1) [225]. The lncRNA ANRIL (antisense non-coding RNA in the INK4 locus), also known as CDKN2B-AS (CDKN2B Antisense RNA 1), protects against hypoxia-induced cardiac injury through the miR-7-5p/SIRT1 axis [226]. The lncRNA HIF1A-AS1 (HIF1A Antisense RNA 1) mediates the expression of SOCS2 (suppressor of cytokine signaling 2) by miR-204 to encourage ventricular remodeling followed by myocardial ischemia/reperfusion injury [227]. The lncRNA CARL (cardiac apoptosis-related lncRNA) has been shown to have a protective role in mitochondrial fission and CM apoptosis during ischemia–reperfusion [228]. Consequently, more investigations are needed to check whether the overexpression of CARL could prevent DOX-induced cardiotoxicity by inhibiting apoptosis and mitochondrial fission, which leads to the improvement of cardiac function. The lncRNA CAREL (cardiac regeneration-related lncRNA) is known to be capable of regulating the proliferation of human iPSC-derived CMs, specifically in the context of cardiac regeneration after injury [229]. Overexpression of lncRNA HOTAIR (HOX transcript antisense intergenic RNA) protects CMs from hydrogen peroxide-induced apoptosis [230].

#### lncRNAs associated with increased risk of cancer therapy-indued cardiotoxicity

##### SOX2-OT (SOX2 overlapping transcript)

The lncRNA SOX2-OT, located on human chromosome 3q26.3 and overlaps with SOX2, one of the major regulators of pluripotency, was found to be significantly upregulated upon DOX treatment leading to the apoptosis of primary CMs in rat models [231]. SOX2-OT exerted its biological function by sponging miR-942-5p. DP5 (death protein 5) was shown to be a direct target of miR-942-5p. The expression levels of DP5 become reduced when miR-942-5p is overexpressed, resulting in reduced apoptosis. Overexpression of SOX2-OT and DOX treatment resulted in increased expression of DP5 and lead to increased levels of apoptosis, due to the downregulation of miR-942-5p. Silencing of either SOX2-OT or DP5 and overexpression of miR-942-5p have all been shown to decrease the amount of cleaved caspase-3 in vitro. Similar to these observations, knockdown of SOX2-OT or overexpression of miR-942-5p conferred cardioprotection against DOX-induced dysfunction in vivo, when measured by LVEF and LVFS (left ventricular fractional shortening) [231]. The lncRNA SOX2-OT is highly expressed in embryonic stem cells and has a crucial role in maintaining the pluripotency of self-renewing and undifferentiated embryonic stem cells [232, 233], demonstrating an important role in development. Its dysregulation in disease states has been reported in the following: diabetic complications, mental illness, gastric cancer, breast cancer, esophageal cancer, pancreatic ductal adenocarcinoma, hepatocellular carcinoma, ovarian cancer, lung cancer, laryngeal squamous cell carcinoma, nasopharyngeal carcinoma, cholangiocarcinoma, osteosarcoma, and glioblastoma. It has also been reported to have prognostic value in some of these states [234, 235]. A study reported that variant 7 of SOX2-OT (SOX2OT-V7) increases DOX-induced autophagy through miR-142/miR-22 [236].

The transcription factor interferon-regulatory factor 4 (IRF4) [237] was found to be responsible for increasing the transcriptional activity of lncRNA SOX2-OT. Depending on its abundance, IRF4 forms a homodimer or a heterodimer within promoter regions where it forms complexes with other transcription factors to regulate the target gene expression [238].

##### LincRNA-p21 (long intergenic non-coding (linc) RNA-p21)

DOX-induced cellular senescence leads to the development of cardiovascular dysfunction [239]. Senescence can be triggered by increased production of ROS and oxidative stress. The lincRNA-p21 was upregulated in HL-1 murine CMs treated with DOX. This upregulation of lincRNA-p21 leads to a decrease in cellular proliferation and viability, as well as increased expression of p53 and p16, and decreased telomere length and telomerase activity [200]. Inhibition of Wnt/β-catenin pathway has been reported to be associated with DOX-induced cardiomyopathy [240]. Previous studies demonstrated the role of lincRNA-p21 in reducing β-catenin in cardiac stem cells [241]. Consistent with these observations, DOX-treatment upregulates the expression of lincRNA-p21 and decreases the expression of β-catenin in HL-1 murine CMs. siRNA mediated knockdown of lincRNA-p21 reversed DOX-induced cellular senescence. These effects were demonstrated through increased cellular proliferation and viability, decreased expression of p53 and p16, and increased telomere length and telomerase activity, suggesting the pro-senescent effect of lincRNA-p21. Silencing of lincRNA-p21 increased β-catenin protein levels. DOX significantly decreased mitochondrial transmembrane potential and SOD (superoxide dismutase) activity, while increasing the production of ROS and activation of lipid peroxidation. This DOX-induced oxidative stress-mediated cellular senescence was reversed by the knockdown of lincRNA-p21. Reduction of oxidative stress by silencing of lincRNA-p21 was subsequently abolished by overexpression of lincRNA-p21, indicating that lincRNA-21 regulated oxidative stress plays a key role in DOX-induced cardiac senescence [200]. An additional role of lincRNA-p21 might also be recognized in endothelial cells since it was found to play a vital role in regulating atherosclerosis [242]. The transcription factors ING1b and p53 regulate the expression of lincRNA-p21 [243]. Wnt/β-catenin singling, a protein-coding downstream target of lincRNA-p21, was reported to be involved in the inactivation of P53 [244]. However, another study reported that β-catenin positively regulates the transcriptional activity of P53 [245].

##### CHRF (cardiac hypertrophy-related factor)

The lncRNA CHRF derived from the intron of the DCC (DCC netrin 1 receptor) gene has been shown to be upregulated in DOX-induced heart failure, both in vitro, and in vivo [246]*.* The inhibition of CHRF reduced the myocardial apoptosis caused by DOX-treatment via the TGF-β/Smads and TGF-β/p38 pathways [246]. Additionally, the adenovirus-mediated overexpression of CHRF reversed the protective effects of valsartan, an angiotensin II receptor blocker, in a murine model of DOX-induced cardiotoxicity [246]. These findings suggest the CHRF might have important roles in multiple tissues of the cardiovascular system. It is also reported that CHRF acts as an endogenous sponge of miR-489 and regulator of MYD88 expression [247]. The CHRF binding site with miR-489 is highly conserved between species, even though the full-length sequence of CHRF is not conserved [247].

##### HOX-AS3 (HOXB cluster antisense RNA 3)

The expression of HOX-AS3 was found to be upregulated in prostate cancer and H9C2 cells upon DOX treatment, where it causes reduced cell viability. The silencing of HOX-AS3 restored the proliferative abilities of CMs in the presence of DOX. A negative correlation was identified between the expression of miRNA-875-3p and HOXB-AS3, suggesting a potential regulatory mechanism. DOX treatment which increases the expression of HOX-AS3, also results in decreased levels of miR-875-3p in CMs. Similarly, the downregulation of miR-875-3p has been observed in other contexts of cardiovascular dysfunction, including children with primary dilated heart disease [248]. Previous studies have also reported the function of HOXB-AS3 as being involved with tumor progression, and the encoded polypeptide can act as a promising anti-tumor drug candidate [249–251].

##### H19

The highly conserved lncRNA H19 is upregulated in myocardial tissue with dilated cardiomyopathy (DCM) induced by DOX [178, 252]. The exact function of H19 in the heart is not well known. However, inhibition of H19 reduced DOX-induced injury in CMs and led to improvements in cardiac functions [253]. The miR-675 is located within the gene of H19. Like the expression of H19, miR675 is also upregulated in DOX-induced cardiotoxicity. Interestingly though, overexpression of miR-675 reverses the protective effect of H19, resulting in CM injury mediated by EBP1 (ErbB3-binding protein 1) [252–254]. In contrast to its role in promoting apoptosis during DOX-induced cardiotoxicity, H19 has been observed to mediate anti-apoptotic effect in a streptozotocin-induced diabetic rat model, indicating that apoptotic characteristics of H19 are specific to stress conditions [255]. The transcription factor E2F1 positively regulates the expression of H19 via binding with promoter [256].

##### PVT1 (plasmacytoma variant translocation 1)

The lncRNA PVT1 is related to a family of oncogenes that have a key role in cardiovascular diseases. PVT1 has been found to be upregulated in CMs treated with DOX [257–259]. Another recent study demonstrated that PVT1 promoted vascular ECs proliferation in CHF by suppressing the activity of miR-190a-5p [260]. PVT1 has also been reported to enhance atrial fibrosis via the miR128-23p-Sp1-THF-β1-Smad axis [261]. The knockdown of PVT1 reduced the DOX-induced cardiotoxicity mediated by miRNA-875-3p and miRNA-187-3p/AOX1 pathways [248, 259]. The transcription factor NFκB1 mediates many of the downstream events related to DOX-induced cardiotoxicity, which ultimately leads to the activation of inflammatory pathways and apoptosis. Inhibition of NFκB1 protects against DOX-induced cardiotoxicity. NFKB1 has binding sites in the promoter region of PVT1, and overexpression of NFKB1 upregulates PVT1 in CMs [262]. A recent study demonstrated that salvianolic acid A reversed DOX-triggered apoptosis in CMs by inhibiting NFKB1 expression, leading to downregulation of PVT1. All these observations were identified in vitro. Therefore, further experiments need to be performed in vivo to reveal the therapeutic potential of this lncRNA since it might regulate several signaling pathways in myocardial toxicity [262]. A study reported that YAP1 positively regulates the expression of PVT1 through direct interaction [263].

##### TINCR (terminal differentiation-induced non-coding RNA)

Pyroptosis is one of the proinflammatory conditions controlled by pyroptotic caspases, and it is now extensively recognized as a key player in the progression of cardiovascular diseases [264]. The characteristics of pyroptosis are characterized by increased inflammation and activation of NLRP3 (NLR family pyrin domain containing 3) along with caspase-1,3,4 and 11, leading to the cleave of GSDMD (gasdermin D) or GSDME and release of IL-1β and IL-18 due to the rupture of the plasma membrane [34]. DOX has been shown to activate pyroptosis in CMs [265]. DOX-treated dying cells showed altered morphological features, including swelling and rupture of cell membrane with increased expression of NLRP3, cleaved caspase-1, IL-1β, IL-18, and GSDMD-N. Treatment of CMs with NLRP3 (NLR family pyrin domain containing 3) inhibitor, MCC950 abrogated the effects of DOX-induced cell death through pyroptosis [266]. Meng et al. established a rat model of myocardial injury followed by treatment with DOX, which showed a decline in LVEF and FS, increases in serum myocardial enzymes including AST, LDH, and CK-MB, as well as increases in pyroptosis markers including NLRP3, cleaved caspase-1, IL-1β, IL-18, and GSDMD-N [267]. The expression of TINCR is significantly increased in CMs and heart tissues of rats followed by DOX treatment. In line with DOX-induced pyroptosis, overexpression of TINCR resulted in declined LVEF and FS, CM damage, and pyroptosis-related proteins, indicating a potential role of TINCR in DOX-mediated proptosis. Knockdown of TINCR offset some of these DOX-induced CM pyroptosis effects resulting in decreased expression of NLRP3, cleaved caspase-1, IL-β, IL-18, and GSDMD-N in myocardial tissues. Studies revealed that DOX increases H3K27ac at the promoter region of TINCR, leading to enhanced expression. Mechanistically TINCR mediates CM pyroptosis through binding with IGF2BP1 (insulin-like growth factor 2 mRNA binding protein 1), which regulates mRNA stability [268]. Consistent with previous findings, TINCR modulates DOX-induced CM pyroptosis by stabilization of NLRP3 mRNA through IGF2BP1 [267]. The role of TINCR was found to be specific in DOX-induced pyroptosis or apoptosis in cardiac cells but not in DOX-induced pyroptosis or apoptosis of cancer cells [267].

STAT3 has binding sites in the promoter region of lncRNA TINCR. A study reported that STAT3 promotes the transcriptional expression of TINCR through a positive feedback loop mechanism via STAT3-TINCR-EGFR axis [269]. Other study reported that IGF2BP1 is involved in regulating STAT3 expression [270], suggesting the possible positive feedback regulation between the upstream transcription factor of TINCR and its downstream targeted protein-coding gene. However, new mechanistic studies are necessary to check this hypothesis. Likewise, a recent study also reported the role of STAT3 in regulating the effects of NLRP3, which is one of the downstream protein-coding genes of lncRNA TINCR [271].

##### LINC00339

The lncRNA LINC00339 was highly upregulated in DOX-induced cardiotoxicity leading to the enhanced apoptosis of CMs. Silencing of LINC00339 showed an anti-apoptotic effect, which rescued the DOX-induced reduction of CM viability. The 3′ UTR of LINC00339 has binding sites for miR-484. Mimics or inhibitor of miR-484 effectively increased or decreased the expression of LINC00339, respectively. These findings indicate that LINC00339 directly targeted miR-484. Similarly, knockdown of LINC00339 resulted in increased expression of miR-484, leading to a reduction in cellular apoptosis and enhanced cellular proliferation, indicating that LINC00339 and miR-484 establish an axis in regulating apoptosis in the DOX-induced cardiotoxicity and that LINC00339 might be involved in cardiac remodeling [272]. The miR-482 has been reported to have a key role in apoptosis and mitochondrial fission mechanisms [272]. Previous reports showed that LINC00339 acts as a precursor of miR-539-5p, and so many descriptions of miR-539-5p functions might also be assigned to LINC00339.

Other lncRNAs that may induce cardiac damage: MEG3 mediated inhibition of miR-7-5p is involved in mediating effects that arise from myocardial ischemia or reperfusion injury [273]. Knockdown of pro-fibrotic lncRNA (PFL) can attenuate cardiac interstitial fibrosis and improve cardiac function. A proposed mechanism of PFL involves a ceRNA mediated regulation of TGF-β1 by sponging let-7d [274].

### miRNAs

**Fig. 3:**
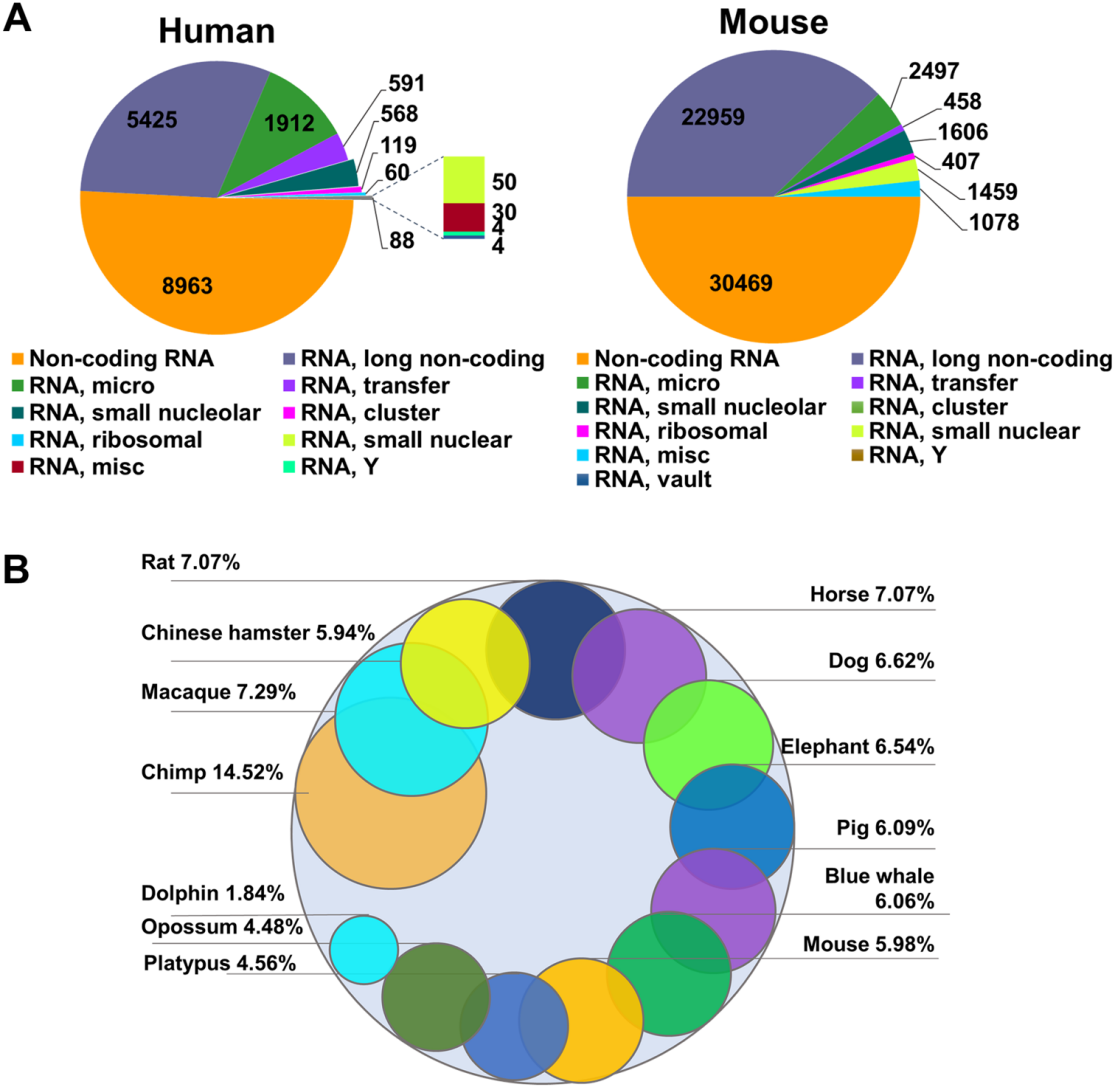
**A** Number of different classes of ncRNAs in human (https://www.genenames.org/download/statistics-and-files/) and mouse (http://www.informatics.jax.org/marker/). **B** Percent of ncRNAs conserved across lineages. Conservation analysis was performed using g:Profiler

**Fig. 4:**
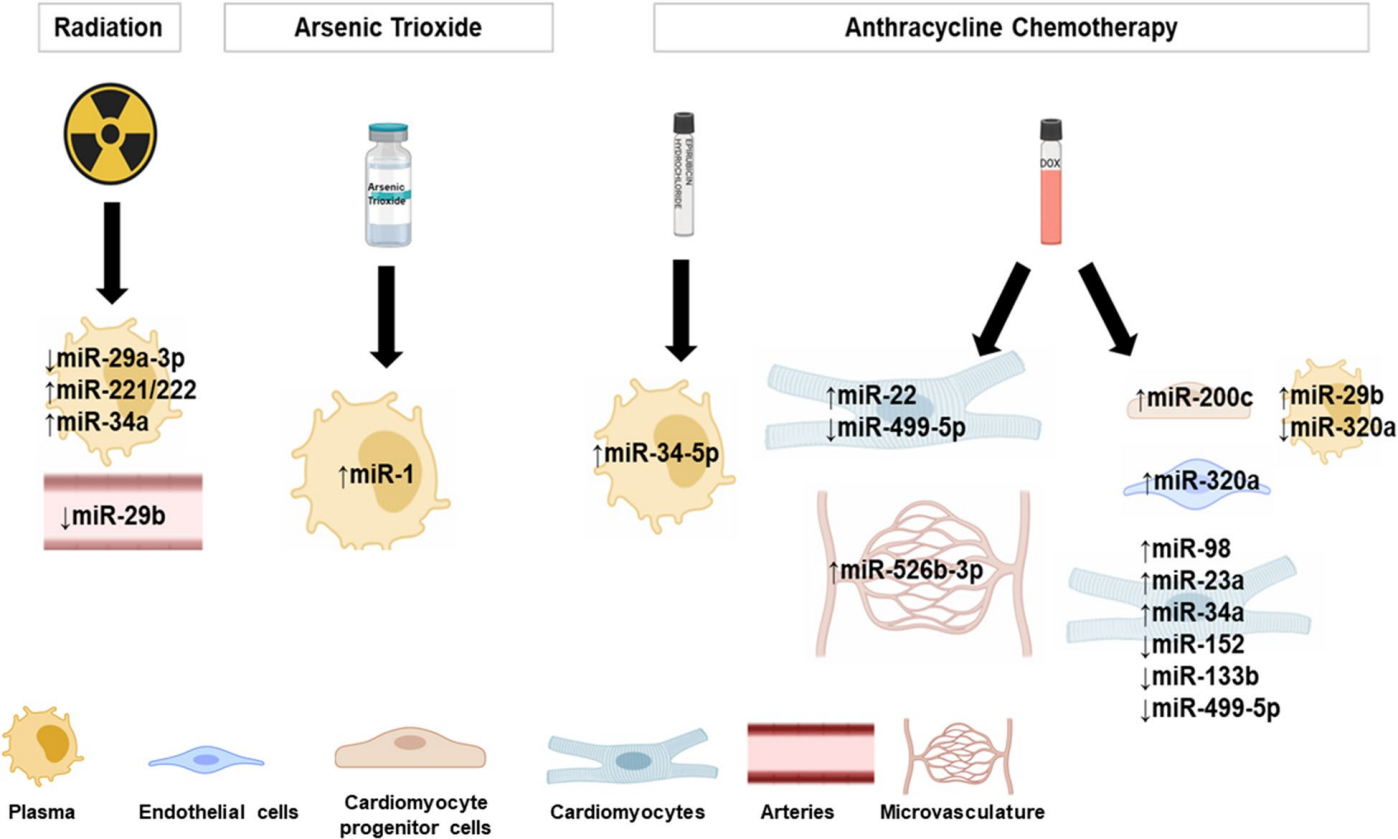
Differentially regulated miRNAs in cardiotoxicity induced by different cancer therapeutic strategies

miRNAs are about 20 nucleotides of non-coding RNAs with a variety of functions, including cell differentiation, proliferation, gene expression, apoptosis, and cancer pathology [275, 276]. Some miRNAs can also be used as a biomarker for cardiac irregularities [277]. They bind to various regions of the mRNA, including the 5′ and 3′ untranslated regions, coding regions, and promoters, targeting both coding and non-coding mRNA post-transcriptionally for degradation and translational inhibition [278]. The miRNAs, like lncRNAs, are encoded in the organism’s genome; however, they do not have as many nucleotides. In humans, there are 1912 miRNA genes (Fig. 3A), and roughly 25% of mRNA genes are believed to be regulated by miRNAs [279]. In cancers, miRNAs commonly act as oncogenes or tumor suppressors. Oncogenic miRNAs overexpressed in cancers are known to target and downregulate tumor suppressors, resulting in cell proliferation and enhanced the strength of the tumor [280]. Simultaneously, some miRNAs interfere with different proteins present in the cell to suppress oncogenic activity [280]. As shown in Fig. 4, miRNAs are affected by different cancer treatments. The differential expression, individualized to each miRNA, may affect the occurrence of cardiotoxicity. In the emerging field of cardio-oncology, the roles of miRNAs in cancer therapy-induced cardiotoxicity are increasingly being noted (Table 2).

**Table 2:** miRNAs involved in cancer therapy-induced cardiotoxicity

miRNA	Orthologs	Drug or type of chemotherapy	Expression	Targets	Cell type	Biological effect	Ref.
miR-152	*Homo sapiens* *Mus musculus* *Rattus norvegicus* *Danio rerio*	DOX	down	NRF2	CMs	Apoptosis, oxidative damage, myocardial inflammation	[281]
miR-133b	*Homo sapiens* *Mus musculus* *Rattus norvegicus*	DOX	down	PTB1, TGLN2	CMs In vivo	Apoptosis, cardiac fibrosis	[293]
miR-98	*Homo sapiens* *Mus musculus* *Rattus norvegicus*	DOX	Up	Fas, RIP3	CMs	Apoptosis	[357]
miR-499-5p	*Homo sapiens* *Mus musculus* *Rattus norvegicus* *Danio rerio*	DOX	Overexpression	P21	CMs and Heart	Improved CM hypertrophy and cardiac function	[358]
miR-29b	*Homo sapiens* *Mus musculus* *Rattus norvegicus*	DOX	Down	Bax, BCL2	CMs and myocardium	Apoptosis, mitochondrial membrane depolarization	[305]
miR-29a-3p	*Homo sapiens* *Mus musculus*	Radiation	Down	Unknown	Secreted exosomes	Cardiac fibrosis	[306]
miR-215-5p	*Homo sapiens* *Mus musculus*	DOX	Up	ZEB2	CMs	Apoptosis	[309, 310]
miR-200c	*Homo sapiens* *Mus musculus* *Rattus norvegicus* *Danio rerio*	DOX	Up	ZEB1	Cardiac mesenchymal progenitor cells	Cardiac progenitor cell depletion	[314]
miR-22	*Homo sapiens* *Mus musculus* *Rattus norvegicus*	DOX	Up	SIRT1	CMs	Apoptosis, oxidative stress	[359]
miR-34a-5p	*Homo sapiens* *Mus musculus*	Epirubicin	Up	SIRT1	Myocardium and plasma	Apoptosis, heart failure	[317]
miR-1	*Homo sapiens* *Rattus norvegicus*	As_2_O_3_	Up	KCNJ2, ERG	CMs	impaired CM electrophysiology	[321]
miR-320a	*Homo sapiens* *Mus musculus* *Rattus norvegicus*	DOX	Up	VEGF-A	ECs	Apoptosis	[322]
miR-526b-3p	*Homo sapiens*	DOX	Up	CD31, CD34, STAT3	ECs	Abnormal capillaries microvasculature, vascular homeostasis	[323]
miR-23a	*Homo sapiens* *Mus musculus*	DOX	Up	PGC-1α	CMs	Mitochondrial injury, apoptosis	[324]
miR-221/222	*Homo sapiens* *Mus musculus* *Rattus norvegicus*	Radiation	Up	c-KIT	ECs	Angiogenesis	[330, 331]

#### Protective miRNAs in cancer therapy-induced cardiotoxicity

##### miR-152

miR-152, a highly conserved miRNA in both rats and humans, was found to be downregulated in DOX-induced cardiotoxicity both in vivo and in vitro [281]. Increased expression of miR-152 reduced DOX-induced cardiotoxicity by attenuating apoptosis of CMs, oxidative damage, and myocardial inflammation, leading to the prevention of cardiac dysfunction. Similar to lncRNA MHRT, the cardioprotective nature of miR-152 was dependent on the activation of the NRF2 signaling pathway [281]. Further experiments need to be performed to check whether overexpression of miR-152 reduces the therapeutic potential of DOX in suppressing cancerous tumors. The upregulation of miR-152 was found to be involved in the development of heart failure [282], whereas increased expression of miR-152 in the neonatal heart helped to maintain energy demands by upholding glycolysis [283].

##### miR-133

The miR-133 family comprises miR-133a-1, miR-133a-2, and miR-133b. This family plays a key role in the pathophysiological processes of heart diseases [284, 285]. miR-133b has antitumor potential as well and is associated with TKI induced cardiotoxicity, cardiac fibrosis, and myocardial infarction [286–290]. Furthermore, miR-133b has also been shown to be capable of serving as a serum biomarker for cardiac fibrosis [291]. miR-133b has been described as having a cardioprotective role in morphine-preconditioned rat CMs [292]. The levels of miR-133b are significantly downregulated in DOX-treated rat ventricular CMs and cardiac tissues [292]. Consistent with these findings, Li et al. reported the decreased expression of miR-133b followed by DOX-treatment in both in vitro and in vivo mice cardiotoxicity models. In a mouse model of DOX-induced heart failure, the levels of BCL2 were downregulated, and the levels of BAX and cleaved caspase-3 became elevated. Overexpression of miR-133 inhibited DOX-induced apoptosis and cardiac fibrosis while increasing the expression of BCL-2 and decreasing the expression of BAX and cleaved caspase-3, collagen I, III, and IV, and fibronectin both in vivo and in vitro. PTB1 and TGLN2 serve as downstream targets of miR-133b. Overexpression of PTBP1 or TAGLN2 reversed the protective effects of miR-133 [293], indicating that miR-133 protects against DOX-induced apoptosis and cardiac fibrosis by inhibiting the expression of PTB1 and TAGLN2. Therefore, miR-133 may serve as a potential biomarker in the diagnosis and treatment of DOX-induced cardiotoxicity, leading to the development of DCM.

##### miR-98

Let-7/miR-98 family members include let‐7: a, b, c, d, e, f, g, i and miR‐98. This family can be considered as the first identified mammalian miRNAs. DOX upregulates miR-98 in CMs. Surprisingly, overexpression of miR-98 was found to be protective against DOX-induced cardiotoxicity by upregulation of caspase-8 and downregulation of Fas and RIP3 [294]. Previous studies showed that miR-98 inhibits apoptosis by targeting caspase-3 [295]. miR-98 has been observed to be downregulated in heart tissues of mice after acute myocardial infarction, as well as H_2_O_2_-treated neonatal rat ventricular myocyte. The overexpression of miR-98 was found to be protective against CMs apoptosis by regulating the Fas/Caspase-3 singling pathway [296]. In contrast to these observations, miR-98 upregulation in cardiac progenitor cells following hydrogen peroxide treatment has been reported to lead to increased apoptosis. Inhibition of miR-98 was found to be protective against cardiac progenitor cell injury through regulation of STAT3 [297]. The confounding patterns of miR-98 expression regarding having either protective or detrimental roles in the context of cardiovascular function might be owed to its participation in a conserved stress response. Thioredoxin 1, for instance, has been shown to attenuate angiotensin II-induced cardiac hypertrophy by increasing the expression of miR-98 [294]. Interestingly though, the upregulation of miR-98 has been recognized in angiotensin II-induced cardiac hypertrophy. An alternative explanation is that miR-98 might have different biological functions and different downstream mechanisms in certain conditions or cell types. Previous studies reported that miR-98 could act as a potential biomarker for the diagnosis of atherosclerosis and cardiac hypertrophy [298]. Upregulation of miR-98 inhibits collagen deposition in human cardiac fibroblasts via downregulation of TGFBR1 [299]. Overexpression of miR-98 downregulated DAPK1 (death-associated protein kinase 1), leading to the attenuation of cardiac ischemia–reperfusion injury [300].

##### miR-499-5p

miR-499-5p becomes downregulated following DOX treatment [301]. miR-499-5p normally targets the cyclin-dependent kinase inhibitor 1a, also known as p21. Targeting of p21 by miR-499-5p prevents the induction of p21 induced mitochondrial fission and myocardial apoptosis [301]. Wan et al. showed this by demonstrating that overexpression miR-499-5p can reduce p21 activity, leading to the aforementioned consequences [301].

##### miR-29b

Ionizing radiation also downregulates specific miRNAs. The family of miR-29, normally anti-angiogenic, interferes with mRNA coding for collagen and matrix proteins involved in cardiovascular fibrosis [302, 303]. The downregulation of miR-29b has been observed to facilitate angiogenesis. The effects of miR-29 in this context were mediated through MAPK/ERK and PI3K/Akt signaling pathways [304]. The study done by Chen et al. emphasized that the maintenance of miR-29b is crucial in a variety of additional cellular pathways as well [304]. Another study done by Jing et al. showed that the in vitro overexpression of miR-29b lessened the severity of the effects of DOX. This was the result of mir-29b targeting the 3′ untranslated region of Bax to increase Bcl-2 expression [305]. The undisturbed Bax and Bcl-2 proteins normally function to cause mitochondrial membrane depolarization and cytochrome *c* release, but due to the downregulation of Bax, this process was inhibited [305]. miR-29b had a protective effect against DOX-induced myocardial apoptosis via a mitochondria-dependent pathway involving Bax [305].

##### miR-29a-3p

Radiation therapy also downregulates miR-29a-3p, a finding which correlates with an increased risk of cardiac fibrosis [306]. There are three types of miR-29 (a, b, and c) in the family, and their sequences vary by only a couple of base pairs [307]. miR-29a-3p was successfully used as a biomarker to detect the amount of radiation that a patient received [306]. miR-29a-3p has also been observed to render cancer cells more susceptible to certain toxicities, improving the efficacy of chemotherapeutic agents to target and treat abnormal cells [308].

#### miRNAs associated with increased risk of cancer therapy-induced cardiotoxicity

##### miR-215-5p

Studies have reported that DOX upregulates miR-215-5P in vitro and in vivo [309, 310]. Mechanistic studies performed in vitro showed that the depletion of miR-215-5p expression alleviated DOX-induced CM apoptosis by upregulating ZEB2 (zinc finger e-box binding homeobox 2). Previous studies demonstrated the role of miR-215-5p in multiple cancers [311]. However, there is a lack of knowledge if miR-215-5p may act as a promising therapeutic target in reducing cardiotoxicity as current evidence is limited to in vitro studies. More in vivo experiments are needed to make conclusive remarks.

##### miR-200

miR-200c comes from the miR-200 family, which plays a role in the epithelial to mesenchymal transition in tumor cells, an important feature of metastasis [312]. DOX upregulates miR-200c, which downregulates ZEB1 (zinc finger e-box binding homeobox 1), endothelial nitric oxide synthase, Sirtuin1 (SIRT1), and Forkhead boxO1, leading to epithelial dysfunction and DOX-related cardiotoxicities such as cardiomyopathy, cardiac apoptosis, and CHF [312, 313]. Stromal cell-derived factor-1 (SCDF-1) can prevent the upregulation of miR-200c. Injections of SCDF-1 into mouse hearts have been observed to be protective against the development of cardiomyopathies resulting from DOX treatment by preventing miR-200c upregulation [314].

##### miR-22

miR-22 is commonly found in cardiac and skeletal muscle and is upregulated in DOX-treated cells [315]. The main target of miR-22 was the 3′ untranslated region of the SIRT1 gene, which leads to the downregulation of SIRT1 [315]. SIRT1 is a deacetylase that targets regulatory proteins and transcription factors that are capable of altering various cellular processes and pathways [316]. The enhanced function of these proteins due to the downregulation of SIRT1 potentially addresses many functions affected by DOX-induced cardiotoxicity. Recent studies demonstrated that the inhibition of miR-22 reduced apoptosis and oxidative stress in CMs [315]. Therefore, specifically targeting miR-22 may be effective in decreasing DOX-induced cardiotoxicity.

##### miR-34-5p

Various cancer treatments result in the upregulation of miR-34-5p [317]. Epirubicin specifically caused an upregulation of miR-34-5p, which targeted SIRT1 for post-transcriptionally mediated downregulation [317]. SIRT1 has also been recognized as inhibiting hyaluronan synthase 2 (HAS2) expression by targeting HAS2-AS1 (HAS2-Antisense RNA 1) [318]. Downregulated HAS2 reduces the production of hyaluronan (HA). HA is one of the key components of endothelial glycocalyx, and diminished production of HA might lead to the degradation of endothelial glycocalyx, resulting in the development of cardiovascular vascular complications [319]. Further understanding of underlying mechanisms of lncRNA HAS2-AS1 or miR-34-5p in degradation of endothelial glycocalyx may provide an avenue for developing novel therapeutic strategies for reducing cardiovascular complications. The miR-34-5p mediated downregulation of SIRT1 also activated the p66-shc pathway [317]. Activation of this pathway caused BAX, and caspase-2 to be upregulated and activated, causing CM apoptosis and heart failure [317, 320].

##### miR-1

The upregulation of miR-1 due to As_2_O_3_ facilitates the downregulation of KCNJ2 post-transcriptionally. KCNJ2 functions as a K+ channel, and its dysregulation contributes to impaired CM electrophysiology [321]. Upregulation of miR-1 also repressed the expression of ERG [321]. The combination of these events caused arrhythmia, prolonged QT intervals, and hypertrophy. The therapeutic potential in targeting miR-1 was demonstrated, whereby knockdown of miR-1 with antisense was shown to inhibit the development of QT prolongation [321].

##### miR-320a and miR-526b-3p

The use of DOX mediates the upregulation of miR-320a [322]. Upregulation of miR-320a has been shown to induce apoptosis, leading to abnormalities in the heart vessels [322]. The miR-320a targeted VEGF-A, a growth factor involved in maintaining homeostasis through vessel formation [322]. Without VEGF-A, the cardiac vessels formed improperly, leading to apoptosis in vitro and vessel abnormalities. To rescue DOX-induced cardiotoxicity, miR-320a knockdown and reintroduction of VEGF-A in cultured ECs restored proliferation activities and prevented apoptosis [322]. DOX had a similar impact on miR-526b-3p as it did on miR-320a [323]. The miRNA was upregulated, causing a decrease in CD31 and CD34, which showed a decrease in the density of the venules, arterioles, and capillaries microvasculature [265]. STAT3, a transcription factor for VEGF-A, was downregulated as a result of upregulated miR-526b-3p [323]. The decrease in STAT3 caused a decrease in the production of VEGF-A [323]. Recent studies showed that in human umbilical vein endothelial cells (HUVECs), the knockdown of miR-526b-3p enhanced tube formation [323].

##### miR-23a

miR-23a is a miRNA normally involved in regulating angiogenesis. In response to treatment with DOX, miR-23a becomes upregulated, causing mitochondrial injury and apoptosis in the CMs [324, 325]. Therapeutic potential for this miRNA was demonstrated with knockdown experiments of miR-23a in DOX-treated CMs, which reduced apoptosis and oxidative stress in CMs [324]. A recent study revealed that the peroxisome proliferator-activated receptor-gamma coactivator-1α (PGC-1α)/Dynamin-related protein (DRP1) pathway was capable of preventing CM apoptosis in this context to promote survival [324]. miR-23a inhibits PGC-1α, a regulator of mitochondrial biogenesis and an inhibitor of DOX-induced cardiomyopathy [324, 326].

##### miR-221/222

Following radiotherapy, the anti-angiogenic miR-221/222 was upregulated, contributing to the radiation-induced cardiovascular dysfunction and cardiac hypertrophy [327, 328]. miR-221/222 targeted c-KIT post-transcriptionally, leading to impairments in angiogenesis [329]. Additionally, the role of miRNA-221/222 in ECs has been recognized as having control over senescence. When upregulated, this then leads to apoptosis and cell death; however, in smooth muscle cells, the upregulation promotes proliferation [330, 331]. Recent studies showed that the knockdown of miR-221/222 contributes to fibrosis and left ventricular stiffness [332].

## Future perspective and conclusions

Despite many years of investigations, the precise mechanism of cancer therapy-induced cardiotoxicities remains poorly characterized. There is an urgent need for new investigations to protect the heart following anticancer therapies. Today, there are a few cardioprotective strategies available to address these concerns. Existing therapies include the use of Dexrazoxane, ACE-inhibitors, angiotensin II receptor blockers (ARB), and beta-blockers. However, these therapies are not in routine prophylactic use and have varying degrees of efficacy. Clinical use of Dexrazoxane to treat anthracycline-induced cardiotoxicity caused significant side effects, such as suppression of activities in the bone marrow [333, 334]. Therefore, novel therapeutic strategies are required to fight against the cardiotoxicity derived from TKIs, anthracycline, and other chemotherapies. The current ongoing research in the field of oncology should be focused on investigating the mode of action of anticancer therapeutic drugs in both cancerous and cardiac cell types to avoid the potential cardiotoxic side effects in patients while retaining the effectiveness of cancer therapies. Moreover, there is an unmet need for interdisciplinary studies in the field of cardio-oncology.

Since ncRNAs emerged as a key regulator of several pathophysiological signaling pathways, they can be promising therapeutic options in the framework of cardio-oncology. ncRNAs in circulation also present an opportunity for novel diagnostic and prognostic markers of disease. Most of the previous studies have been performed by using a limited number of subjects. Hence, clinical studies should be performed with a large-scale randomized and controlled subject in determining the potential biomarkers for cardiotoxicity. Differentially regulated ncRNAs can be targeted using multiple methods, including adeno-associated viruses (AAV), nanoparticles, antagomir, GapmeR, and locked nucleic acid, use of small molecular inhibitors targeting the inhibition of lncRNA-RNA-binding protein interactions, genome-editing using CRISPR/Cas9, knockdown of respective natural antisense transcripts or degradation of lncRNAs located in the cytoplasm by using a siRNA-based approach involving the multiprotein complex RISC, RNAse dicer, endonuclease Argonaut2-dependent degradation pathway, and chemically modified antisense oligonucleotides (ASOs) resulting in RNAseH-dependent degradation [335–338]. The therapeutic use of ASOs for nuclear-localized lncRNAs has its limitations since ASO-mediated cleavage of nascent RNAs can induce premature termination of transcription. A major issue in the therapeutic targeting of ncRNA is how cardioprotection might be achieved without interfering with the effects of anticancer drugs or disease progression. Effective and novel methods need to be developed for therapeutic manipulations of dysregulated lncRNAs and miRNAs to reverse or prevent anticancer drug-induced cardiotoxicity.

Since ncRNA research has consistently progressed over the past decade, more complex tools need to be developed to modulate the expression of lncRNAs or miRNAs to establish novel strategies to counteract the cardiotoxicity of anticancer drugs. Among all kinds of ncRNAs, miRNAs are the most extensively studied in understanding cancer therapy-induced cardiotoxicity. Recent findings suggest that lncRNAs can be used as a biomarker for detecting cardiovascular complications. lncRNAs can also act as promising therapeutic targets as they are stably expressed and tissue-specific. However, further investigations are needed to determine if lncRNAs can act as a diagnostic tool for the early prediction of anticancer therapy-induced cardiotoxicity. Novel, cutting-edge technologies will deliver a new avenue for in-depth analysis of cardiac function during cancer treatments which might recognize opportunities for treatments that impact the delicate balance of ncRNAs present by recognizing pathways with dysregulated RNAs.

Although lncRNAs are promising therapeutic targets, some of the translational studies in animal models are difficult due to their poorly conserved sequences between species. Consequently, only the highly conserved lncRNAs may act as therapeutic targets for novel therapies. It is estimated that over 80% of human lncRNAs are non-conserved. Hence, the physiological function of lncRNAs cannot be effectively studied in animal models. It is worth noting that despite low sequence conservation, lncRNAs have higher tissue specificity (78%) than miRNAs (19%), which may provide important clues about their specific therapeutic functions within a specific tissue or cell type [104]. Forthcoming novel studies need to be focused on developing a valid in vivo humanized animal model that can be used to study the non-conserved human lncRNAs specific to the heart. As per our knowledge, a liver-specific humanized mouse model is currently being used for validating the physiological function of liver-specific lncRNAs [339].

More investigations are needed to identify un-annotated lncRNAs because there is no single lncRNA therapeutic approach performed in large animal models to date. A study focused on analyzing lncRNAs from three farm animals, chicken, cattle, and pigs, revealed that half of the identified lncRNAs were not annotated in NCBI or related databases [340]. Moreover, lncRNAs from these species were less conserved. The experiments and observations generated by using in vitro systems and small animal models such as rodents are drawbacks in developing innovative therapeutic strategies. Most have failed to replicate the same results in larger animal models. Rodents have a lot of fundamental differences from larger animals and humans, especially in cardiovascular physiology. Before starting the clinical application, proof of concept and safety evaluation studies must be conducted in larger animal models.

Even though the data generated from large animal models are more promising for human diseases, there are certain limitations in working with larger animal models, including expensive maintenance, large breeding space, time-consuming experimental procedures, longer gestation time, and difficulties associated with the generation of gene knockin/knockout models. Utilizing the most advanced human engineered heart tissues (EHTs) and living myocardial slice models derived from human cells or tissue might solve these limitations, and it may act as an alternative to larger animal models.

The focus on ncRNA-based therapeutic approaches is gradually increasing in recent years from the beginning of approval of a small interfering RNA drug called patisiran in 2018 by the FDA. This drug degrades mRNA coding for transthyretin in polyneuropathy [341]. Since the expression of lncRNA and miRNA vary from one area to another area within the same tissue over time with concomitant conditions, ncRNA-based therapy remains in its early stages. However, further strategies need to be developed for tissue- and cell type-specific delivery, which targets only deregulated ncRNAs with reduced off-target effects in innate immune responses.

Though lncRNAs can act as a sponge of miRNAs, the underlying mechanisms, or downstream targets of sponged miRNAs in the context of cancer therapy-induced cardiotoxicity remains not well understood. Additional studies are needed to delve deeper into this interplay as downstream targets of sponged miRNAs can also serve as therapeutic targets. As it is beyond the scope of this current review to provide a list of predicted miRNAs that can be sponged, investigators can take advantage of available databases such as LncCeRBase [342], lncRNASNP2 [343], DIANA-LncBase v3 [344], LncMirNet [345], lnCeDB [346], SomamiR 2.0, [347], miRSponge [348], and starBase V2.0 [349].

In the future, we will continue to observe the aberrantly expressed ncRNAs to determine their exact function and impact on cardiovascular health, as well as the adverse effects that anticancer drugs have on patients. Previous literature has demonstrated that TKI inhibitor sunitinib causes severe cardiovascular complications, but the role of ncRNAs in mediating sunitinib-induced vascular toxicity which may lead to the development of cardiovascular complications remains unknown. Forthcoming studies need to be focused on this area to develop a novel ncRNA-based therapeutic approach. The research in the field of ICI-induced cardiotoxicity is still in the early stages; more investigations may need to be performed to identify dysregulated ncRNAs. Similarly, the current research focus on the cardiotoxicity of CAR T-cell immunotherapy is very limited, and preventive strategies to minimize cardiovascular complications remain vague. Therefore, forthcoming investigations must be focused to understand the role of ncRNAs in CAR T-cell immunotherapy-induced cardiotoxicity. In conclusion, novel studies on ncRNAs to reveal early detection of chemotherapy-induced cardiotoxicity will be crucial in the use of different chemotherapeutic agents in clinical settings and might be of use in the development of therapeutic strategies to address the needs of cardio-oncology.

## Data Availability

Not applicable.
